# Small coastal streams—Critical reservoirs of genetic diversity for trout (*Salmo trutta* L.) in the face of increasing anthropogenic stressors

**DOI:** 10.1002/ece3.6306

**Published:** 2020-05-17

**Authors:** R. Andrew King, Bruce Stockley, Jamie R. Stevens

**Affiliations:** ^1^ Department of Biosciences College of Life and Environmental Sciences University of Exeter, Hatherly Laboratories Exeter UK; ^2^ Westcountry Rivers Trust Callington UK

**Keywords:** bottleneck, habitat fragmentation, microsatellite, migration barriers, *Salmo trutta*

## Abstract

We used microsatellite markers to investigate levels and structuring of genetic diversity in trout (*Salmo trutta* L.) sampled from 16 rivers along the south coast of Cornwall in southwest England. This region is characterized by many small coastal streams with a few larger catchments. At a regional level, genetic structuring of contemporary populations has been influenced by a combination of events, including the last Ice Age and also more recent human activities over the last millennium. All populations are shown to have gone through strong genetic bottlenecks, coinciding with increased exploitation of mineral resources within catchments, beginning during the Medieval period. At more local levels, contemporary human‐induced habitat fragmentation, such as weir and culvert construction, has disproportionally affected trout populations in the smaller catchments within the study area. However, where small catchments are relatively unaffected by such activities, they can host trout populations with diversity levels comparable to those found in larger rivers in the region. We also predict significant future loses of diversity and heterozygosity in the trout populations inhabiting small, isolated catchments. Our study highlights how multiple factors, especially the activity of humans, have and continue to affect the levels and structuring of genetic diversity in trout over long timescales.

## Graphical abstract

Structuring and levels of genetic diversity in brown trout populations along the south Cornwall stretch has been influenced by multiple natural and anthropogenic factors. At a regional scale, factors since the last glacial maximum have left their imprint. At finer local scales, the main driver of evolutionary change appears to be human activity. This area of southwest Britain has a long history of mining for metal ores. This mining would have affected fish populations in many ways, including the construction of weirs to divert water to ore processing mills and the release of mine water contaminated with heavy metals into rivers.

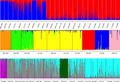

## 1 INTRODUCTION

Identifying patterns of intraspecific genetic diversity and investigating the processes that generate such patterns is a central tenet of molecular ecology and conservation genetics. It is clear that both historical events, such as the Quaternary glaciations (Bernatchez & Wilson, 1998; Jenkins, Castilho, & Stevens, 2018) and more recent anthropogenic impacts (Dufresnes et al., 2018; Waters & Grosser, 2016), have had an influence on levels and patterns of variation. Such factors affect the interplay between the key processes of gene flow, drift, and selection in determining the patterns and levels of variation seen in present day populations. Moreover, in small populations—whether wild (Keller & Waller, 2002), managed, for example, zoo populations (Frankham, 2010), or domesticated (Bruford, Bradley, & Luikart, 2003)—the effects of these factors are typically amplified, leading to increasingly rapid losses of genetic diversity through fixation due to drift or selection. In a study of particular relevance to the topic, Spielman, Brook, and Frankham (2004) explored the impact of genetic factors on extinction risk for threatened populations and species and articulated the idea that reduced population genetic diversity correlates with reduced reproductive fitness and an elevated risk of future extinction linked to genetic factors. More generally, Spielman et al. (2004) linked the degree to which a population is threatened with population size, with small populations being more likely to be classified as threatened than large populations.

Freshwater habitats are among some of the world's most threatened, suffering from five major human‐mediated threats, namely over‐exploitation, pollution, modification of flows, habitat degradation, and the spread of invasive, non‐native species (Dudgeon et al., 2006). These anthropogenic impacts directly affect the physio‐chemical conditions within freshwater habitats and, consequently, have strong influences on the aquatic biota within these habitats (Schinegger, Trautwein, Melcher, & Schmutz, 2012) affecting biodiversity at multiple levels. However, while in freshwater systems—as in their terrestrial counterparts—the strength of the effect of these human impacts on intraspecific diversity and differentiation will be dependent on population size and magnitude of selection (Einum, Fleming, Cote, & Reynolds, 2003; Frankham et al., 2017), the size of a river catchment will also play a potentially critical role, with populations in smaller catchments being particularly at risk (Consuegra, Verspoor, Knox, & García de Leániz, 2005; Whelan, 2014). Moreover, there are a number of specific threats from which fish populations in small streams are likely to suffer more than larger rivers. Of particular interest are issues with access for anadromous fish species, connectivity between small streams and larger catchments or the sea, the impact of barriers to fish movement and water flow fluctuations (Griffiths, Koizumi, Bright, & Stevens, 2009; Palm, Laikre, Jorde, & Ryman, 2003; Thorstad, Økland, Aarestrup, & Heggberget, 2008). Barriers, both natural and man‐made, can impact rivers by dividing continuous habitat into smaller patches (Jones et al., 2019). From a genetic perspective, this subdivision can have multiple adverse effects on fish populations. Barriers that prevent movement of fish between habitat patches can result in reductions in both census and effective population sizes and increased inbreeding, which, in turn, can lead to reduced levels of genetic diversity, and an increase in genetic structuring (Frankham et al., 2017; Griffiths et al., 2009; Montgomery et al., 2000; Palm et al., 2003).

Anadromous salmonid species, known for their strong homing fidelity to their natal rivers (Keefer & Caudill, 2014), are often found in spatially structured metapopulations (Schtickzelle & Quinn, 2007). This philopatry is highly adaptive, increasing the likelihood that fish will find suitable spawning and juvenile habitats (Keefer & Caudill, 2014) and giving rise to the aforementioned spatially structured metapopulations. However, straying of fish to non‐natal rivers also occurs (King, Hillman, Elsmere, Stockley, & Stevens, 2016; Valiente, Beall, & Garcia‐Vazquez, 2010) and is recognized as an important evolutionary feature of salmonids, especially in range expansion and colonization of newly open habitats, and can facilitate gene flow between rivers (Horreo et al., 2011; Keefer & Caudill, 2014).

The role of small streams (for which there is no universally accepted definition; Biggs, Nicolet, Mlinaric, & Lalanne, 2014) in the ecology and population genetics of brown trout (*Salmo trutta* L.) is not well understood (Thomson & Lyndon, 2018; Whelan, 2014). There is a disproportionately large number of these streams in southwest England, where the Devon/Cornwall peninsula precludes the development of large dendritic catchments and it has been suggested that the relatively small populations of trout residing in such small streams may collectively make a significant contribution to the genetic diversity of the species, particularly in relation to those fish exhibiting an anadromous (sea trout) life cycle (Consuegra et al., 2005; Whelan, 2014), albeit with the caveat that small populations also increase the effects of genetic drift, often leading to distinct but genetically depauperate populations (e.g., Paris, King, & Stevens, 2015; Perrier, Ferchaud, Sirois, Thibault, & Bernatchez, 2017). Indeed, while these coastal streams may not be good holding habitats for resident trout, they may offer significant areas of spawning habitat for anadromous fish that are able to access them.

In general, small populations are more likely to suffer from the detrimental effects of genetic bottlenecks, inbreeding, and genetic drift. These processes can lead to the loss of genetic diversity and inbreeding depression (Vandewoestijne, Schtickzelle, & Baguette, 2008). Ultimately, this loss of genetic diversity can lead to a reduction in individual fitness and an increased risk of local population extinction (Vandewoestijne et al., 2008). The effects of genetic drift and inbreeding can be counteracted by both gene flow from other populations and mutation. Over recent timescales (i.e., since the end of the Quaternary glaciations), however, insufficient time has elapsed and mutation rates are generally too slow for mutation to have contributed significantly to increasing levels of genetic diversity (Ho & Larson, 2006). Gene flow, therefore, is the only process that can be relied upon to maintain diversity within small populations. For gene flow to be effective, there has to be a high degree of connectivity between populations, which can be a major problem for populations inhabiting rivers with significant barriers to upstream migration.

In this paper, we explore the genetic structure of—and connectivity between—trout populations in small streams and larger catchments along the south coast of southwest England. This coast is characterized by a few larger catchments and numerous small streams, and resident trout were sampled from both for genetic analysis. This approach allows the genetic structure of small populations of coastal stream trout along this section of coast to be set in a wider context and enables us to address the relative importance of population size and demographic factors in shaping contemporary patterns of genetic variation in trout in small streams.

## 2 MATERIALS AND METHODS

### 2.1 Sample collection

**FIGURE 1:**
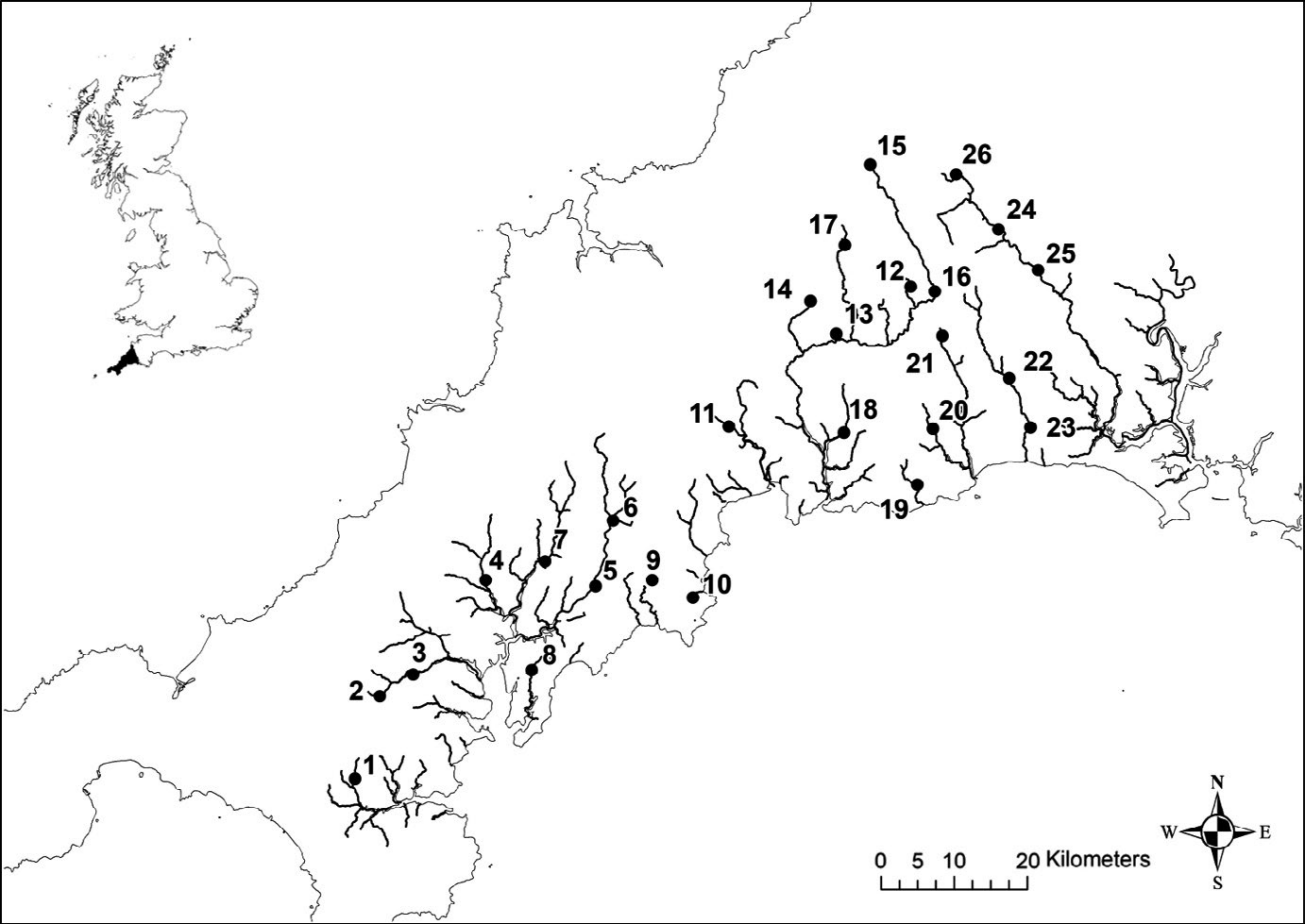
Map showing the sampling location of *Salmo trutta* in southwest England. Inset shows Britain with Cornwall highlighted in black. Full site details are given in Table 1

Individual brown trout were sampled from 26 sites from 16 rivers and streams on the south Cornwall coast between Falmouth Bay in southwest Cornwall and the Tamar estuary in east Cornwall (Table 1, Figure 1, Table A1). Fish were caught during routine Environment Agency electrofishing surveys. The sampling scheme employed was designed to reduce the collection of potentially related individuals by targeting 1+ or older fish. However, in some sample locations where fish densities were low, fry were also sampled to increase sample sizes (and were later excluded from the analysis if found to be part of a full‐sib group—see below). Fish were anaesthetized using MS‐222 prior to removal of adipose their fin using sharp scissors. Fin clips were transferred immediately into tubes containing absolute ethanol. Genomic DNA was extracted from fin tissue following the method of Truett et al. (2000).

**TABLE 1:** Details of samples of trout analyzed and results for basic measure of genetic diversity (*H*_O_—Observed heterozygosity, *H*_E_—expected heterozygosity, *A*_r_—allelic richness, *P*_A_—number of private alleles, *F*_IS_—inbreeding coefficient)

Site	Code	River	Tributary	Location	Grid reference	*n*_1_	*n*_2_	*H*_O_	*H*_E_	*A*_r_	*P*_A_	*F*_IS_
1	HEL.MER	Helford	Main river	Merther Uny Mill Gweek	50.1159, −5.2130	40	37	0.654	0.658	6.95	3	0.020
2	KEN.TRE	Kennall	Main river	Tregolis (upstream)	50.1803, −5.1837	41	35	0.669	0.686	6.93	1	0.040
3	KEN.PON	Kennall	Main river	Ponsvale (downstream)	50.1984, −5.1470	47	42	0.748	0.732	9.1	4	−0.005
4	ALL.DAU	Allen	Main river	Daubauz's Moor	50.2720, −5.0550	50	47	0.763	0.761	10.81	1	0.009
5	FAL.TGY	Fal	Main river	Tregony	50.2674, −4.9186	47	43	0.792	0.793	11.52	2	0.013
6	FAL.TRE	Fal	Main river	Trenowth	50.3191, −4.8993	36	34	0.767	0.763	10.93	0	0.009
7	TRE.GEE	Tresillian	Main river	Geen Mill	50.2864, −4.9816	48	45	0.756	0.778	11.44	4	0.039
8	PER.TRE	Percuil	Main river	Trevinnick Meadow	50.2015, −4.9986	46	44	0.620	0.600	6.05	1	−0.022
9	CAE.KIL	Caerhays	Main river	Kilbol	50.2718, −4.8501	50	48	0.746	0.767	11.63	5	0.038
10	POR.GAL	Portmellon	Main river	Galowras Mill	50.2577, −4.8002	50	38	0.679	0.674	6.27	2	0.012
11	PAR.BRI	Par	Main river	Bridges Moor	50.3919, −4.7560	41	36	0.693	0.704	9.14	8	0.029
12	FOW.BUL	Fowey	Main river	Bulland Farm	50.4975, −4.5013	50	43	0.776	0.764	10.18	0	−0.003
13	FOW.CAB	Fowey	Main river	Cabilla Wood	50.4643, −4.6221	44	38	0.783	0.785	11.76	1	0.016
14	FOW.CAR	Fowey	Cardinham	Cardinham Bridge STW	50.4896, −4.6531	40	37	0.782	0.771	10.92	2	−0.010
15	FOW.LES	Fowey	Main river	Leskernick	50.5996, −4.5739	39	36	0.771	0.770	10.19	1	0.004
16	FOW.TRE	Fowey	Trenant	Wortha/Carpuan	50.5005, −4.5310	50	49	0.774	0.784	11.18	3	0.023
17	FOW.WAR	Fowey	Warleggan	Temple	50.5337, −4.6118	48	43	0.786	0.781	10.56	0	0.006
18	LER.COL	Lerryn	Main river	Collon	50.3871, −4.6122	50	49	0.782	0.809	12.39	3	0.043
19	POL.LON	Polperro	Main river	Longcoombe Mill	50.3459, −4.5228	50	44	0.761	0.753	7.97	0	0.002
20	WLO.GIL	West Looe	Main river	Gillhill Wood	50.3895, −4.5026	50	49	0.790	0.803	12.68	4	0.026
21	ELO.HIG	East Looe	Main river	Highwood	50.4626, −4.4910	49	46	0.809	0.807	12.3	1	0.009
22	SEA.COU	Seaton	Main river	Courtneys Mill	50.4284, −4.4089	35	34	0.789	0.764	9.77	2	−0.017
23	SEA.HES	Seaton	Main river	Hessenford	50.3902, −4.3831	36	35	0.770	0.776	10.54	1	0.022
24	LYN.BAT	Lynher	Main river	Bathpool	50.5451, −4.4205	50	45	0.771	0.793	11.45	2	0.039
25	LYN.KER	Lynher	Main river	Kerney Mill	50.5137, −4.3723	48	43	0.765	0.776	10.81	1	0.026
26	LYN.KNI	Lynher	Main river	Knighton	50.5880, −4.4736	39	37	0.811	0.788	10.78	1	−0.016

Note

*n*_1_—sample size; *n*_2_—sample size after removal of full‐sibs and salmon × trout hybrids.

Abbreviation: STW, sewage treatment works.

### 2.2 Microsatellite genotyping

Samples were screened for variation with 18 sets of nuclear microsatellite markers: SsosL311, SsosL417 (Slettan, Olsaker, & Lie, 1995), SsaF43 (Sánchez et al., 1996), BG935488, CA048828, CA060208, CA060177 (Vasemägi, Nilsson, & Primmer, 2005), SSsp2213 (Paterson, Piertney, Knox, Gilbey, & Verspoor, 2004), Ssa407UOS (Cairney, Taggart, & Høyheim, 2000), One102 (Olsen, Wilson, Kretschmer, Jones, & Seeb, 2000, using the primers of Keenan et al., 2013), SsaD58 and SsaD157 (King, Eackles, & Letcher, 2005), sasaTAP2A (Grimholt, Drabløs, Jørgensen, Høyheim, & Stet, 2002), STR3QUB (Keenan et al., 2013), Ssa85 and Ssa197 (O'Reilly, Hamilton, McConnell, & Wright, 1996), SS11 (Martinez, Moran, & Garcia‐Vasquez, 1999), and BHMS362 (also known as Ssa52NVH (Gharbi et al., 2006); AF256702). Five of the loci (Ssa85, BG935488, CA060208, CA060177, and sasaTAP2A) showed nonoverlapping size ranges between trout and Atlantic salmon (*Salmo salar* L.) and were therefore useful for the identification of trout x salmon hybrids. Polymerase chain reactions (PCRs) and genotyping were performed as described in Paris et al. (2015).

### 2.3 Data quality assurance

Micro‐Checker v 2.2 (Van Oosterhout, Hutchinson, Wills, & Shipley, 2004) was used to detect the presence of large allele dropout, stuttering, and null alleles at each locus. Genepop v 3.4 (Raymond & Rousset, 1995) was used to test for linkage disequilibrium (LD) between all pairs of loci within each population and for deviation from Hardy‐Weinberg Equilibrium (HWE) for each locus and population. Significance was estimated using a Markov chain method (1,000 de‐memorizations, 100 batches, and 1,000 iterations). False discovery rate (FDR, Benjamini & Hochberg, 1995) was used to correct significance levels for all multiple comparisons.

Populations of brown trout can often contain a large number of closely related individuals (Hansen, Nielsen, & Mensberg, 1997). The presence of full‐sibs can potentially lead to bias in population genetic parameter estimation (Goldberg & Waits, 2010) and false inference of population structure (Andersen & Dunham, 2008; Rodríguez‐Ramilo & Wang, 2012). The program COLONY v 2.0.5.9 (Jones & Wang, 2010) was used for sibship reconstruction. This program implements a maximum likelihood method to assign sibship to individuals using their multilocus genotype. To check for consistency of results, the program was run twice using different random number seeds. Conditions were high precision, medium length run, assuming both male and female polygamy without inbreeding, and a 1% error for both scoring error rate and allelic dropout rate. Fish were considered members of a full‐sib family if the probability of exclusion was >0.9. Only a single individual of each full‐sib group was retained in the data set for subsequent analyses.

### 2.4 Measures of genetic diversity

Basic measures of genetic diversity (number of alleles per locus and per population, observed heterozygosity and expected heterozygosity (*H*_O_ and *H*_E_, respectively)) were calculated using GenAlEx v6.502 (Peakall & Smouse, 2006, 2012). Calculation of allelic richness (*A*_R_), estimated using the rarefaction method, was implemented in HP‐RARE 1.0 (Kalinowski, 2005). Mean within‐river relatedness, based on Queller and Goodnight’s (1989) pairwise measure, was calculated in GenAlEx v6.502 (Peakall & Smouse, 2006, 2012). We performed 999 permutations to determine whether mean values were significantly different from zero and 999 bootstraps to estimate the 95% confidence limits of the mean. Levels of genetic differentiation between populations were assessed using Weir and Cockerman's (1984) estimator of *F*_ST_. Global and population pairwise values of *F*_ST_ were calculated using MICROSATELLITE ANALYSER v 4.05 (MSA; Dieringer & Schlötterer, 2003). Significance of *F*_ST_ was assessed by 10,000 permutations of genotypes among populations. For the population pairwise analysis, a Bonferroni correction was applied to account for multiple testing.

### 2.5 Analysis of genetic structure

Two different analyses were used to test for population structure within the data set. Firstly, a model‐based clustering method (STRUCTURE v 2.3.4, Pritchard, Stephens, & Donnelly, 2000) was used to determine the structure of populations. The program uses a Bayesian‐based Markov Chain Monte Carlo (MCMC) approach to jointly define *K*, the number of possible partitions of the data set, and determine the proportion of an individual's genome (*q*) derived from each of the *K* partitions. STRUCTURE was run for 250,000 iterations following a burn‐in of 100,000 iterations with the number of inferred populations (*K*) ranging from one to 12. To identify finer levels of structure, hierarchical analyses were performed based on the optimum *K* value from the first run. Analysis parameters for the hierarchical analyses were as given above except that the maximum *K* was set at *n* + 1, where n represents the number of populations in the analysis. Ten independent runs of the program were performed using the admixture model with correlated allele frequencies and not using the population of origin information. The most likely number of clusters was calculated using the Δ*K* method of Evanno, Regnaut, and Goudet (2005). Consensus data were visualized using POPHELPER v1.0.6 (Francis, 2017). Secondly, discriminant analysis of principal component (DAPC; Jombart, Devillard, & Balloux, 2010) analyses were undertaken using the *adegenet* (Jombart, 2008) package for R (R Core Team, 2018). The optim.a.score() function was used to select the optimum number of principal components to be retained in the analysis. Results were visualized as scatter plots. Because the STRUCTURE analysis showed there was no evidence of within‐river genetic structuring (see Section 3), the DAPC analysis was conducted on the data for each river rather than individual sampling locations. As with STRUCTURE, we performed hierarchical analyses where distinct “outlier” populations were successively removed from the data set and the DAPC repeated. An outlier population was one that exhibited a somewhat distinct cluster of individuals based on plots of DAPC1 v DAPC2 and DAPC1 v DAPC3.

To test for isolation by distance (IBD), a Mantel test (Mantel, 1967) was used to evaluate the relationship between linear genetic [*F*_ST_/(1−*F*_ST_)] and geographical distances (km) between populations (Rousset, 1997). Pairwise geographic distances between river mouths were calculated using the R package *marmap* (Pante & Simon‐Bouhet, 2013). Distances from river mouths to each sampling site were calculated using an online distance calculator (http://www.daftlogic.com/projects‐google‐maps‐distance‐calculator.htm). The Mantel test was performed with 999 permutations using GenAlEx v6.502 (Peakall & Smouse, 2006, 2012).

### 2.6 Bottleneck analyses

Evidence for the presence of genetic bottlenecks in each of the sampled rivers and groups identified in the STRUCTURE analyses was assessed using both the heterozygote excess (Cornuet & Luikart, 1996) and M‐ratio (Garza & Williamson, 2001) methods. The program BOTTLENECK (Piry, Luikart, & Cornuet, 1999) was used to test for an excess of heterozygotes against expectations for a population at mutation‐drift equilibrium under the two‐phase mutation (TPM) model of microsatellite evolution. We set the proportion of multistep mutations in the TPM model to 20% and the variance of TPM to 30%. Significance was tested using the Wilcoxon's sign‐rank test for a one‐tailed heterozygosity excess. Allele frequency distributions were also examined to determine whether mode shifts had occurred. We also calculated M, the ratio of the number of alleles at a given microsatellite locus to the allelic size range for that locus (Garza & Williamson, 2001). Following the suggestion of Peery et al. (2012), we set the proportion of multistep mutations and the mean size of multistep mutations to 0.22 and 3.1, respectively. Critical *M* (*M*_c_), the expected value of *M* in a population at mutation‐drift equilibrium, was calculated based on multiple values of the prebottleneck effective population size (50, 100, 250, 500, and 1,000) and a mean mutation rate (*µ*) of 5 × 10^–4^ (Paris et al., 2015) such that Θ (4*N*_e_ *µ*) varied from 0.1 to 2.

We also investigated long‐term changes in effective population size (*N*_e_) using VarEff v1.2 (Nikolic & Chevalet, 2014). The program estimates temporal changes in *N*_e_ using an MCMC approach. Estimates of effective size were generated from sampling time to 300 generations in the past (1,200 years assuming a generation time of 4 years; Jensen et al., 2008). We set the effective size prior to 10,000 and used the two‐phase model of microsatellite evolution with the proportion of multistep mutations set to 0.2 and assuming a mutation rate of 5 × 10^–4^ (Paris et al., 2015). The length of burn‐in was 10,000 steps with data generated from 10,000 batches of length ten with a sampling interval of ten steps giving a total of 10^6^ data points. Data were analyzed for each individual river, and the three multiriver groups identified by the STRUCTURE analysis. VarEff requires microsatellite loci to have three or more alleles. Therefore, the data for locus One102b were removed. Additionally, trout populations in some rivers possessed only two alleles for Ssa85. For consistency, data for this locus were also removed for all rivers and groups.

### 2.7 Gene flow analyses

Two methods were used to estimate historical and contemporary gene flow between rivers. Migrate‐n v3.7.2 (Beerli & Felsenstein, 2001) uses a Bayesian coalescent approach to jointly estimate theta (4*N*_e_ *µ*, where *µ* is the mutation rate) and *M* (*m*/*µ*, where *m* is the migration rate). We used a Brownian motion mutation model with starting parameters based on *F*_ST_ calculations and a constant mutation rate for each locus. We used uniform priors for both theta (min = 0, max = 10) and *M* (min = 0, max = 1,000). To estimate recent migration rates of trout between rivers, we used BayesAss 3.0.4 (Wilson & Rannala, 2003). The program implements a Bayesian method to estimate the proportion of immigrants in a population. The mixing parameters Δ*A*, Δ*F*, and Δ*M* were each set to 0.15. Three runs were performed using 10^7^ iterations (with a burn‐in of 10^6^ iterations) and a sampling interval of 1,000 iterations. Migration rates were calculated as the average of the three runs.

To predict potential future reductions in heterozygosity for each of the 16 sampled rivers, we used the method proposed by Crow and Kimura (1970). Predicted levels of heterozygosity (*H*_t_) at 10, 50, and 100 generations in the future were calculated as:

$$H_{t}=H_{O}{\left(1-\frac{1}{2N_{e}}\right)}^{t}$$

where *H*_O_ is the current observed heterozygosity, *N*_e_ is the current effective population size, and *t* is time in the future in generations. The model assumes random mating and that reductions in heterozygosity are due solely to genetic drift. Effective population size was calculated using two methods: the linkage disequilibrium method as implemented in NEESTIMATOR v.2 (Do et al., 2014), using a minimum allele frequency of 0.02 and the sibship method as implemented in COLONY v 2.0.5.9 (Jones & Wang, 2010).

## 3 RESULTS

### 3.1 Data quality assurance

The 18 pairs of primers amplified a total of 19 loci. The primers for one marker, One102, amplified two loci with nonoverlapping size ranges and were designated One102a and One102b. A total of 1,174 fish were genotyped. Four fish (three from the river Par [PAR.BRI] and one from the East Looe [ELO.HIG]) were identified as trout × salmon hybrids and were removed from the data set. COLONY identified a total of 67 full‐sib families (range 1–6 full‐sib families per sample site, with 2–5 members per family—Table A2). This resulted in the removal of 97 fish leaving a final data set of 1,077 trout on which all further analyses were performed.

Using the program Micro‐Checker, suspected null alleles were detected in 12 loci in 13 populations. Tests for linkage disequilibrium found only 15 out of 4,446 tests were significant after FDR correction. Significant deviations from HWE, after FDR correction, were found for 12 tests comprising eight loci and ten populations. As none of these significant results were consistent across loci or populations, all loci and populations were retained for further analyses.

### 3.2 Measures of genetic diversity

Measures of genetic diversity were generally high across all trout populations. A total of 452 alleles were found at the 19 loci. The number of alleles per locus ranged from two (One102a) to 50 (SsaD58). Allelic richness ranged from 6.06 (PER.TRE) to 12.68 (WLO.GIL; Table 1). Both observed heterozygosity and expected heterozygosity were lowest for trout in the Percuil (PER.TRE, *H*_O_ = 0.620, *H*_E_ = 0.600) and highest in the Lerryn (LER.COL, *H*_O_ = 0.809, *H*_E_ = 0.807; Table 1). A total of 52 private alleles were found. Four populations had no private alleles (Table 1). For the remaining populations, the number of private alleles ranged from one to eight (average 2.32 per population).

Global *F*_ST_ was 0.062 (*p* < .0001) for the full data set and 0.024 (*p* < .0001) for the “core” data set (data removed for six “outlier” small streams—see next section). Population pairwise *F*_ST_ values ranged from 0.0026 (FAL.TGY v FAL.TRE to 0.217 (GWE.MER v PER.TRE). After Bonferroni correction, ten out of 325 pairwise *F*_ST_ comparisons were nonsignificant.

### 3.3 Analysis of genetic structure

**FIGURE 2:**
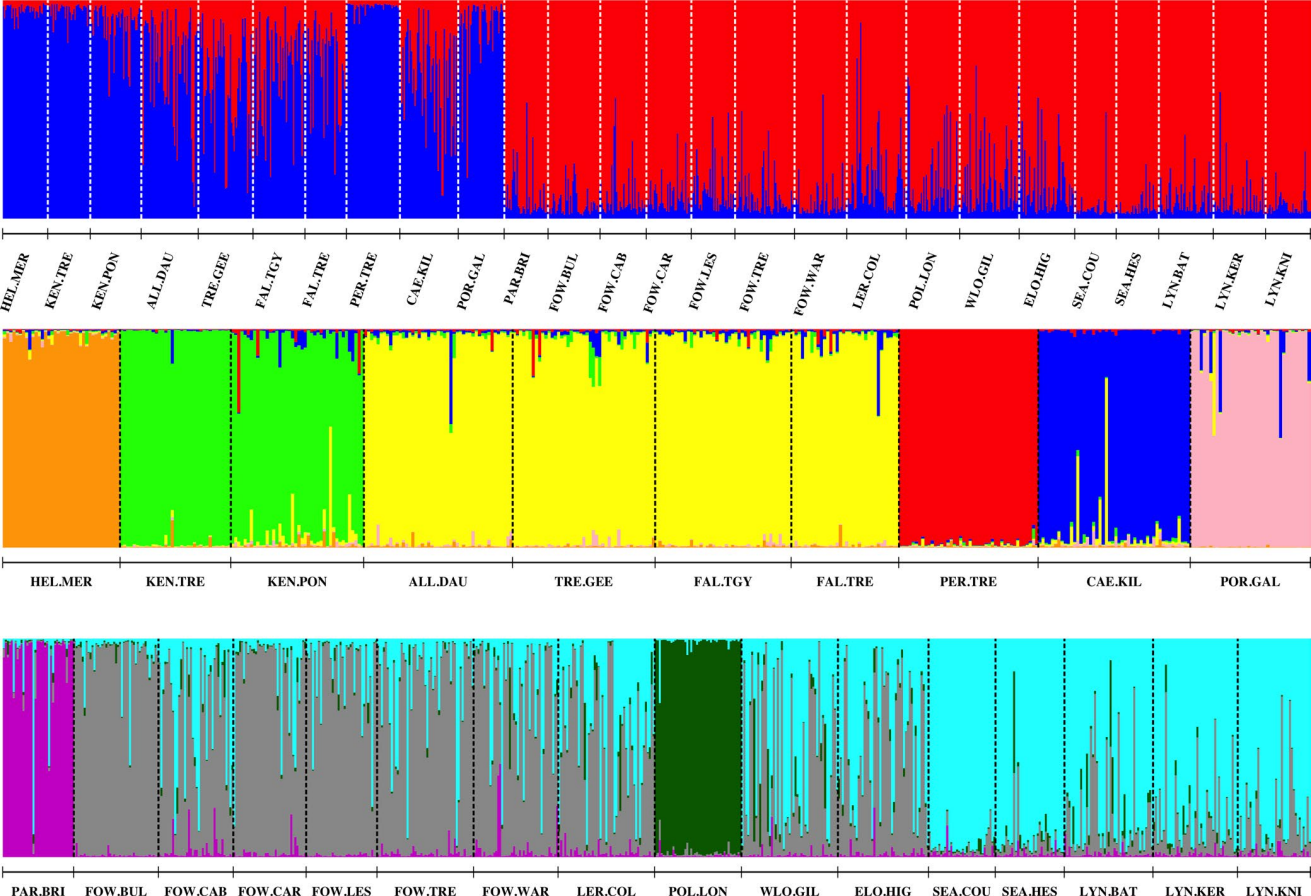
Results of the STRUCTURE analysis for the 26 south Cornwall samples of resident trout: top panel—all populations (*K* = 2), middle panel—western populations (*K* = 6), and bottom panel—eastern populations (*K* = 4)

The STRUCTURE and DAPC analyses were in general agreement, both identifying a group of small “outlier” catchments (Helford River, Kennall, Percuil, Portmellon, Par, and Polperro) and a set of generally larger rivers. For the STRUCTURE analysis, the Evanno Δ*K* method identified *K* = 2 as the most likely partition of the data (Figure 2) splitting the rivers into western (Helford River to Portmellon) and eastern groups (Par to Lynher). Hierarchical analysis of these two groups showed further structure within the data set. For the western group, the optimum *K* was six with the rivers of the Carrick Roads (Allen, Tresillian, and Fal) constituting a group and the other small streams being distinct entities (Figure 2). For the eastern group, *K* = 4 was optimum (Figure 2). Here, trout from the Par and Polperro were distinct, while fish in the remaining rivers showed varying degrees of admixture between two genetic groups. Based on the proportion of these two groups, two sets of populations could be identified, one containing the Fowey, Lerryn, and Looe samples and the other the Seaton and Lynher populations (Figure 2). Thus, ten groupings of trout were identified: Helford River, Kennall, Carrick Roads (CARR), Percuil, Caerhays, Portmellon, Par, Fowey/Lerryn/Looe (FLL), Polperro, and Seaton/Lynher (SELYN).

**Figure A1:**
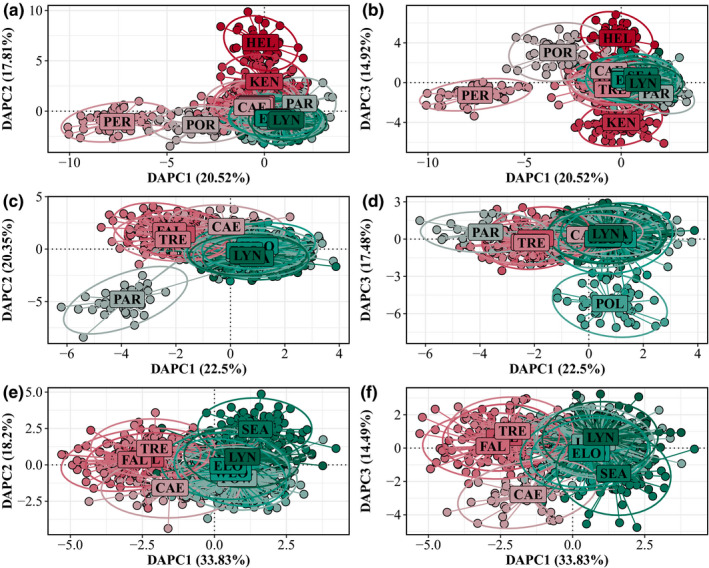
Discriminant Analysis of Principal Components (DAPC) analyses for trout from 16 south Cornish rivers. Each dot represents a sampled individual fish and rivers are coloured in shades of red and green to represent the rivers belonging to the western and eastern groups, respectively, as identified in the STRUCTURE analysis (Figure 2). Scatter plots representing DAPC1 v DAPC 2 (a, c, e) and DAPC1 v DAPC3 (b, d, f). (a and b) scatter plots for all 16 rivers (*N*_inds_ = 1077); (c and d) first hierarchical analysis (*N*_rivers_ = 12, *N*_inds_ = 881), removing samples from the Helford, Portmellon, Percuil and Kennall rivers; and (e and f) second hierarchical analysis (*N*_rivers_ = 10, *N*_inds_ = 801), removing samples from the Helford, Portmellon, Percuil, Kennall, Par and Polperro rivers

As the STRUCTURE analyses showed a lack of genetic structuring of trout within rivers, the DAPC analysis was performed at the river level, combining multiple collections from the same river into a single sample. Successive hierarchical DAPC analyses also showed that six of the small streams were distinct. Analysis of the whole data set showed trout in the Helford, Portmellon, Percuil, and Kennall rivers to be distinct (Figure A1a,b), with the Par and Polperro rivers being distinct in the first hierarchical analysis (Figure A1c,d). The second hierarchical analysis (Figure A1e,f) showed a split between the remaining western and eastern rivers.

**FIGURE 3:**
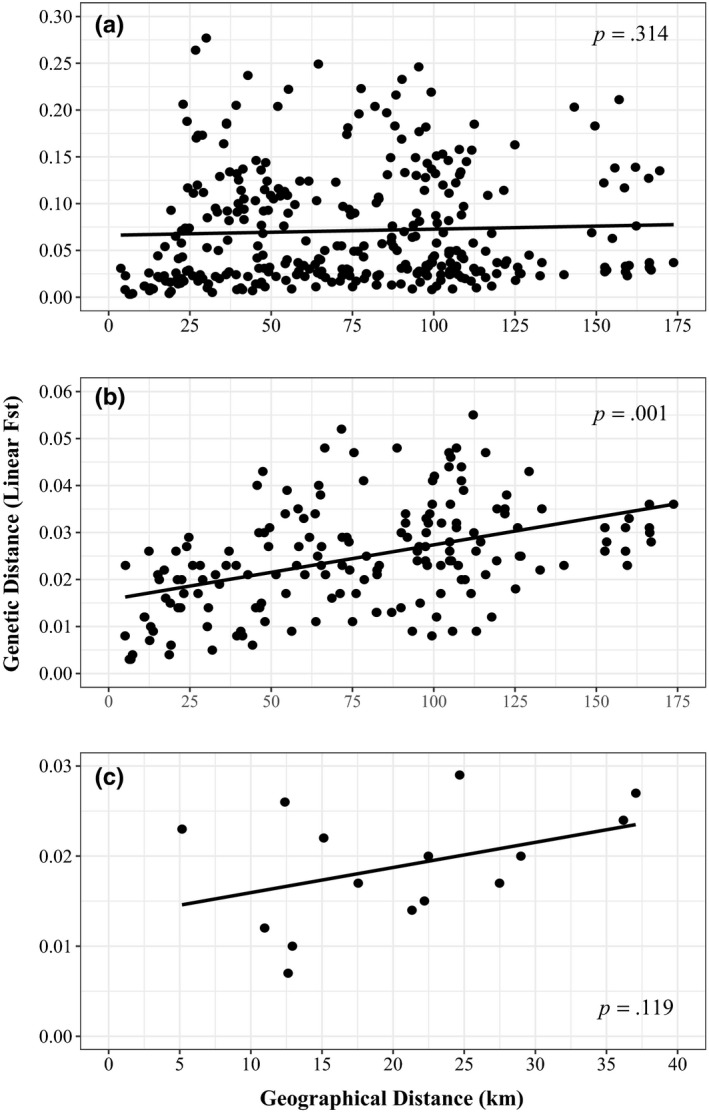
Correlation between geographic distance (km) and genetic distance (linear *F*_ST_) for (a) the full data set (*r* ^2^ = .003, *p* = .327), (b) the larger rivers data set (removing the six outlier small stream populations: *r* ^2^ = .193, *p* = .001), and (c) data for trout from the River Fowey only (*r* ^2^ = .146, *p* = .119)

A Mantel test showed a nonsignificant relationship between geographical and genetic distances for the full data set (Figure 3a, *r* ^2^ = .003, *p* = .327). However, a Mantel test on a data set comprising only the generally larger, nonoutlying populations showed a significant positive correlation between geographic and genetic distances (Figure 3b, *r* ^2^ = .193, *p* = .001). For the River Fowey—the largest river in the study—there was no evidence of IBD between trout populations within the Fowey (Figure 3c, *r* ^2^ = .146, *p *= .119).

**Figure A2:**
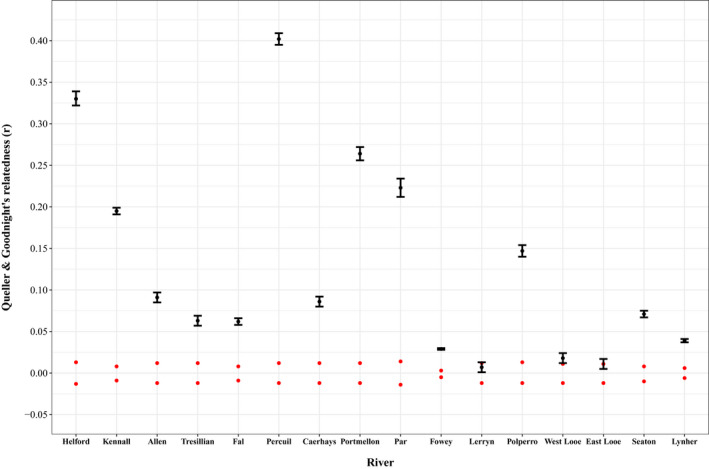
Results for Queller & Goodnight’s (1989) relatedness statistic, as calculated in GenAlEx v6.502. Black dots—mean intra‐population relatedness (±95% CIs); red dots—upper and lower 95% confidence intervals about the null hypothesis of no difference in mean relatedness within rivers as determined by permutation

Mean within‐river relatedness ranged from a minimum of 0.006 for trout from the Lerryn to a maximum of 0.401 for fish from the Percuil (Figure A2). Only the values for the Lerryn and East Looe were not significantly different from zero. There was a clear difference between trout in the six small outlier streams identified in the STRUCTURE and DAPC analyses and the remaining populations, with fish in the outlier rivers showing higher levels of relatedness (mean 0.259 vs. 0.049, minimum 0.146 vs. 0.006, maximum 0.401 vs. 0.088).

### 3.4 Bottleneck analyses

**Figure A3:**
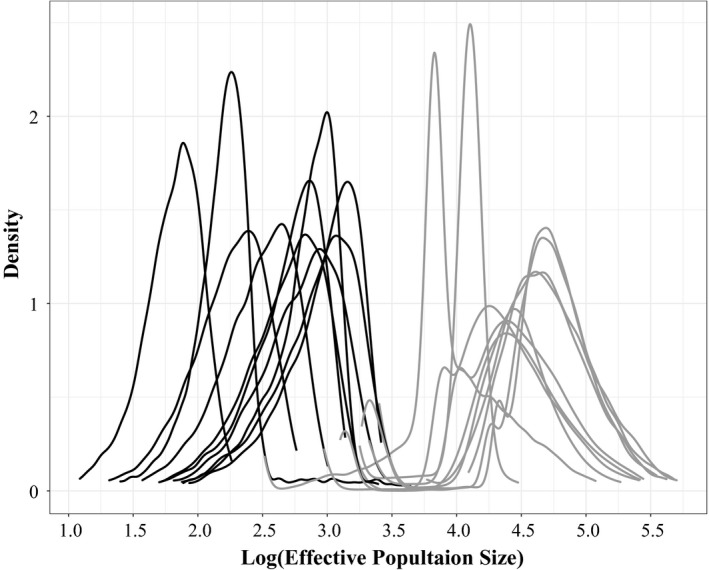
VarEff analysis of the ten population groupings identified from the STRUCTURE analyses. Plots show the posterior density distribution for the log current effective population size (black lines) and for 300 generation ago (grey lines)

The heterozygosity excess method suggested that fish in only a single river, the Polperro, had gone through a recent bottleneck (Table A3). For all rivers, the allele frequency distribution had a normal L‐shaped distribution. However, the M‐ratio test indicated that trout in the majority of rivers and STRUCTURE groupings had experienced a more ancient bottleneck. The exceptions were the FLL and SL groups and the River Fowey, with the SL group and the Fowey failing to show a bottleneck only at Θ values greater than 1 (Table A3). The VarEff results indicated that all rivers and STRUCTURE groupings had gone through severe genetic bottlenecks (Figure A3), typically suffering significant reductions in *N*_e_ over the last 1,200 years.

**FIGURE 4:**
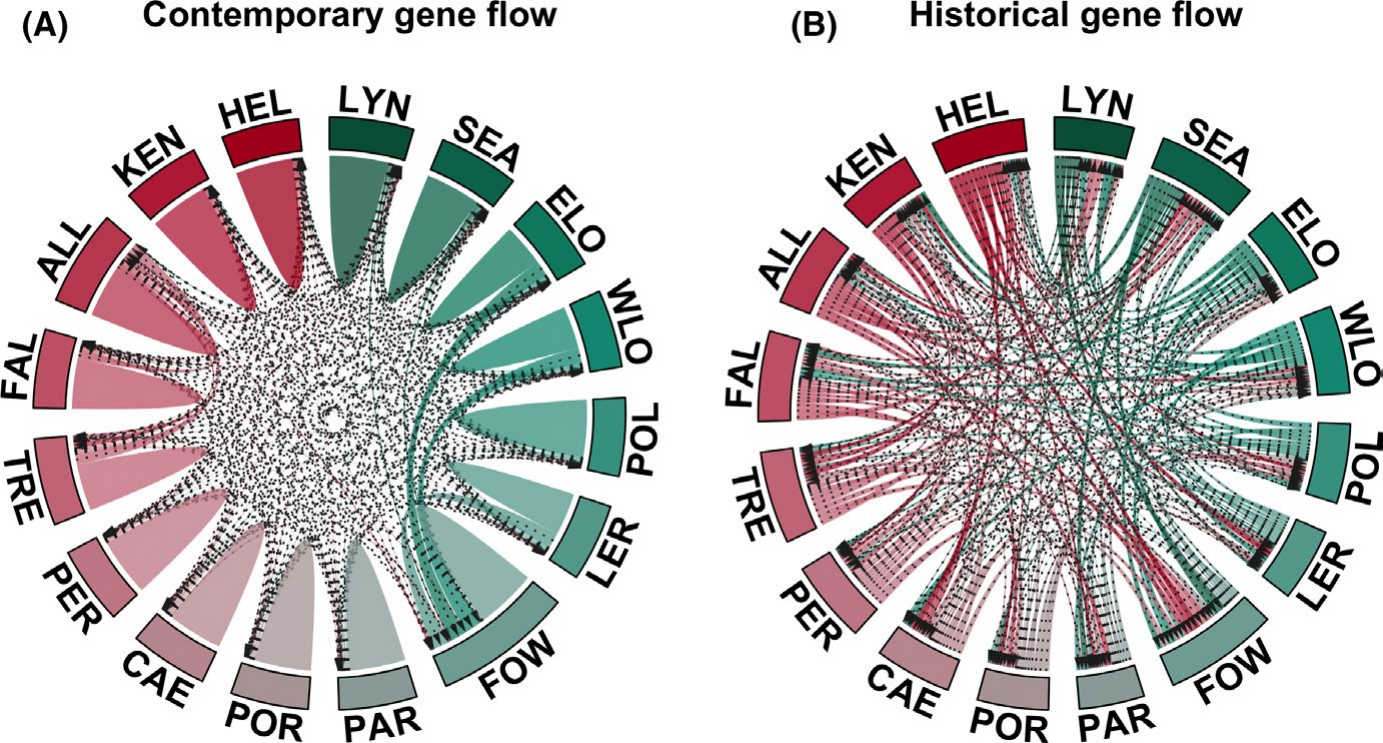
Contemporary and historical gene flow diagrams based on the results of BayesAss and migrate‐n, respectively. Rivers abbreviations are as given in Table 1. Rivers are colored in shades of red and green representing the rivers belonging to the western and eastern groups, respectively, as identified in the STRUCTURE analysis (Figure 2). Arrow direction represents direction of gene flow between rivers. Bumps in the contemporary plot represent self‐assignment of fish to their own river

The BayesAss analysis showed that the majority of individuals analyzed assigned to their river of origin (Figure 4a). Most contemporary gene flow was between rivers within each of the western and the eastern groups of catchments (as identified by STRUCTURE, Figure 2), with little gene flow between the eastern and western groups of rivers (Figure 4a). Indeed, the only notable levels of contemporary gene flow were between the rivers of the Carrick Roads (Fal, Allen, and Tresillian) in the western group and between the Fowey, Lerryn, and East and West Looe in the eastern group. By contrast, the migrate‐n analysis (Figure 4b) showed complex patterns of historical gene flow between rivers, with extensive bi‐directional gene flow not only between rivers within each of the western and eastern groupings but also between the western and eastern groups (Figure 4b).

**FIGURE 5:**
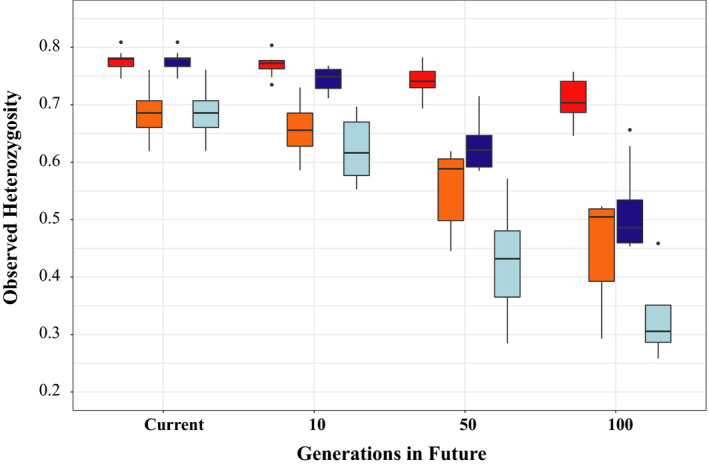
Plot showing predicted future reductions in observed heterozygosity (*H*_O_), compared to current levels based on Linkage Disequilibrium (LD) and sibship‐based estimates of effective population size (*N*_e_). Red and orange boxplots show the predicted reductions in H_O_ for the larger, non‐outlier rivers and the outlier streams (Helford River, Kennall, Percuil, Portmellon, Par, and Polperro), respectively, using LD‐derived *N*_e_, while blue and light blue boxplots show the predicted reductions in H_O_ for non‐outlier and outlier rivers, respectively, using sibship‐derived *N*_e_

Using the approach of Crow and Kimura (1970), the larger rivers within the data set are predicted to lose an average of 8.6% (range 2.8%–14.9%) and 33.56% (range 15.70%–42.01%) of their heterozygosity by 100 generations in to the future, based on the LD and sibship methods of calculating *N*_e_, respectively (Figure 5, Table A4). Conversely, the outlier rivers are predicted to lose an average of 34.5% (range 20.1%–56.9%) and 60.42% (range 33.57%–82.43%) of their heterozygosity, based on the LD and sibship methods of calculating N_e_, respectively, over the same time period.

## 4 DISCUSSION

Analysis of trout from 16 rivers and streams in south Cornwall genotyped at 19 microsatellite loci identified highly contrasting patterns of diversity, relatedness, and genetic differentiation, with six small stream trout populations being distinct from those inhabiting geographically proximal larger catchments.

The initial STRUCTURE and hierarchical DAPC analyses showed a strong regional organization of the genetic diversity in the trout populations analyzed, splitting populations into eastern and western groups. During the last glacial period, sea levels were up to 130 m lower than at present and the English Channel was largely dry land. Southern Britain was not glaciated during the last Ice Age (Clark, Hughes, Greenwood, Jordan, & Sejrup, 2012), and most contemporary rivers and streams would have been the headwaters of much larger rivers that drained through paleochannels into the eastern Atlantic (Antoine et al., 2003). While the courses of these paleorivers are evident in the eastern English Channel (Antoine et al., 2003), the submerged valleys of Cornish rivers (rias) can only be traced close to the current coastline (Camm & Dominy, 1998; Smith, 1989), with the rivers and streams flowing into them once being tributaries of much larger rivers. This is reflected by the close genetic relationships between trout in the main rivers of the Carrick Roads (Fal, Allen, and Tresillian), the Fowey and the Lerryn, and the East and West Looe (Figure 2), suggesting that there are still high levels of contemporary gene flow between populations in the rivers flowing into each of these rias. Presumably, this is mediated either via straying of anadromous sea trout (King et al., 2016) or possibly via movement of nonanadromous individuals through brackish estuarine waters (Taal et al., 2018).

At smaller geographical scales, the genetic structure is often locally complex. Trout populations in the larger catchments and some of the smaller streams display significant isolation by distance, with fish in geographically proximal rivers tending to be genetically similar. However, it is clear that trout in six of the rivers studied here do not fit this pattern and that a mixture of evolutionary, anthropogenic, and environmental processes have acted to alter the levels of genetic diversity and differentiation between them and the generally larger, more “characteristic” rivers of the region. Similar patterns have been found in other fish species. For example, clear regional structure was found in Baltic populations of pike (*Esox lucius*); however, at local levels this structure was more complex (Bekkevold, Jacobsen, Hemmer‐Hansen, Berg, & Skov, 2015). The lack of IBD for the full data set analyzed in the current study suggests that genetic drift is an important process in shaping patterns of genetic variability of trout populations along the south Cornish coast (Hutchison & Templeton, 1999) and this pattern appears to be driven by the marked divergence of fish in six of the smallest catchments. Similarly, a within‐river study of genetic structure in brown trout only showed significant IBD after samples from above impassable barriers were removed from analyses (Griffiths et al., 2009), while Pearse, Martinez, and Garza (2011) found that historic patterns of IBD had been erased in contemporary populations of steelhead (*Oncorhynchus mykiss*) and that this was partly due to fragmentation of rivers by dams.

**Figure A4:**
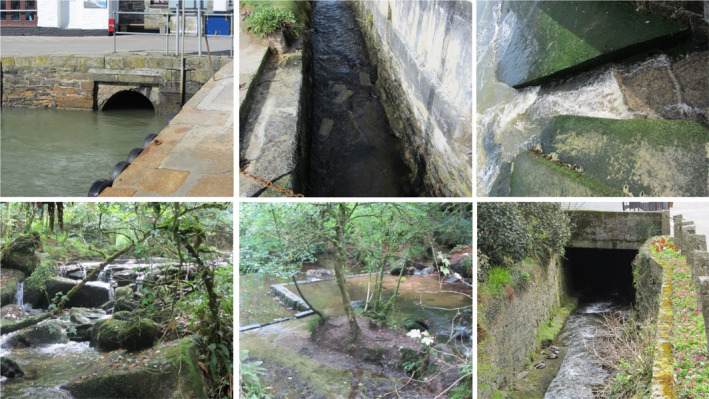
Examples of barriers in four streams in south Cornwall. Clockwise from top left: the mouth of the Mevagissey Stream; a canalised section of the Portmellon stream; the mouth of the Portmellon stream; the Polperro flowing through one of several culverts in Polperro village; heavily silted impounded section of the Kennall River at the Kennall Vale gunpowder works; barrier to upstream movement of trout, Kennall Vale.

There are two main reasons for the distinctiveness of trout in the six outlier rivers. Trout in four of these rivers (the Kennall, Percuil, Portmellon, and Polperro) are probably distinct due to the presence of barriers such as culverts (Portmellon and Polperro) and weirs (Kennall and Percuil; Figure A4). The presence of barriers causes habitat fragmentation which can reduce genetic diversity and increase differentiation in two ways. Firstly, the presence of barriers can prevent movement of fish between suitable habitats both within and between rivers (in the case of trout via straying of anadromous sea trout). Secondly, barriers can reduce the availability of suitable habitat, particularly spawning habitat, leading to reductions in fish population size, which, in turn, can lead to reductions in genetic diversity.

It is often assumed that declines in fish populations are relatively recent and have coincided with the effects of processes such as industrial pollution, over‐fishing and modern, large‐scale river modifications (Lenders et al., 2016). Many studies have shown the effects of this relatively recent human activity on genetic diversity and structure in various fish and aquatic animal and plant species (e.g., Paris et al., 2015). However, it is now apparent that human activities have been affecting fish populations in Western Europe over much longer time scales (Hoffman, 2005; Lenders et al., 2016).

Southwest Britain has a long history of mining, spanning from the prehistoric, through the Roman and Medieval periods to the present day (Gerrard, 1996). Early mining techniques such as tin streaming required huge volumes of water to wash away soil overlaying the metal ore deposits. This resulted in the construction of weirs, leats (artificial water courses), and dams (Gerrard, 1999, 2000) to channel water from rivers and streams to where it was needed for streaming. Up until the Industrial Revolution, subsequent processing of the metal ore relied on mills and smelters powered by waterwheels (Gerrard, 1999) that also required diversion and damming of rivers. However, while it is clear that historic mining has affected the majority of catchments in the area (Bryan & Hummerstone, 1977; Pirrie, Power, Rollinson, Cundy, & Watkins, 2002; Pirrie, Power, Wheeler, et al., 2002), the effects on resident trout populations have been felt most keenly in the smallest catchments. Larger catchments appear able to buffer against localized reductions in fish population size caused by mining activities, perhaps due to the often‐patchy distribution in time and space of mining‐related activities. Knaepkens, Bervoets, Verheyen, and Eens (2004) found that population size was crucial in determining the levels of genetic diversity in Belgian populations of *Cottus gobio*, with larger catchments being able to maintain large population sizes both in terms of census and effective population sizes and, conversely, having lower levels of genetic drift. If there were local reductions in population size (or extinctions) caused by mining activities, these areas could have been readily recolonized by local fish. The lack of IDB in the River Fowey—one of the larger catchments included in this study—would tend to support the ready movement of trout within catchments.

The presence of impassable barriers also threatens the long‐term persistence of populations (Crook et al., 2015; Morita & Yamamoto, 2002) making it unlikely that genetic diversity can be increased naturally through the straying of sea trout into these streams to spawn. Reductions in genetic diversity have been found in many populations of fish isolated above impassable barriers, such as weirs and culverts and natural waterfalls. Populations of coastal cutthroat trout (*Oncorhynchus clarki clarki*) isolated above impassable waterfalls were highly differentiated from downstream populations, having much lower levels of diversity and heterozygosity (Whiteley et al., 2010). Likewise, anadromous *O. mykiss* (steelhead) sampled downstream of dams in the Columbia River were significantly more diverse than resident rainbow trout populations from above the dams (Winans et al., 2018). Likewise, despite the presence of suitable upstream habitat, trout are absent from the Mevagissey stream, a small river close to the Portmellon, that flows into St Austell Bay. It is thought that there has been a local extinction of trout in this stream. However, due to impassable barriers in the lower reaches of the stream, including a 150‐m‐long culvert, natural recolonization of the stream has not occurred.

For the two remaining outlier rivers (the Helford River and the Par), there are no barriers to fish movement between the sampling sites and the sea and their distinctiveness is likely due to the effects of mining activities on their waters and associated polluting effects on the fish populations within them. DiBattista (2008) found that pollution can have contrasting effects on measures of genetic diversity, with some studies showing increases (perhaps due to increased mutation rates or selection for heterozygous individuals) and others decreases (due to decreased population sizes and selection for homozygous individuals) in diversity. Tin streaming and deep rock mining are known to have taken place in the Helford catchment during Medieval times (Dines, 1956). The Par has been adversely affected by waste from the china clay mining industry. Mining for the clay started in the mid‐18th century, releasing large quantities of silt into rivers which would have been particularly detrimental to trout and salmon. Fine sediments can impact fish both directly and indirectly. Suspended sediments can cause damage to gills, increase stress levels, and affect feeding and growth rates of fish (Kemp, Sear, Collins, Naden, & Jones, 2011). Indirectly, sediments can clog up spawning gravels and reduce the amount of dissolved oxygen available to developing eggs (Kemp et al., 2011). Additionally, significant reductions in abundance and diversity of invertebrates on which trout feed have been reported in clay‐affected rivers (Nuttal & Bielby, 1973).

The genetic diversity of trout in the small streams that have been badly affected by mining practices within their catchments may have been further compromised due to a lack of gene flow. Our analyses showed substantial levels of historical gene flow between the rivers analyzed here which contrasted greatly with estimated levels of contemporary gene flow. However, high levels of dissolved metal in river water could act as a chemical barrier to fish movement in much the same way as physical barriers, such as weirs. Laboratory experiments have shown that metal‐naïve fish generally avoid sublethal concentrations of toxic metals (Araújo, Moreira‐Santos, & Ribeiro, 2016; Woodward, Hansen, Bergman, DeLonay, & Little, 1995), while the upstream migration of spawning Atlantic salmon in a tributary of Miramichi River in Canada was affected by high levels of copper and zinc in river water leaching from a mine, with very high levels of metals appearing to completely stop fish movement altogether (Saunders & Sprague, 1967). Similarly, Paris et al. (2015) suggested that reduced movement of fish through a region of high toxic metal contamination is responsible for genetic subdivision of brown trout populations in the River Hayle in west Cornwall.

Together, these multiple processes have resulted in genetic erosion of trout in small streams across the region. Exposure to industrial pollution can elicit multiple possible responses including migration, local extinction, or adaptation (Bijlsma & Loeschcke, 2012). Experimental populations of *Chironomus riparius* rapidly lost genetic diversity when exposed to environmentally relevant concentrations of tributyltin (Nowak et al., 2009), while brown trout populations inhabiting rivers with high levels of heavy metal pollution had significantly lower levels of neutral genetic diversity than fish in corresponding relatively clean rivers (Paris et al., 2015). However, responses to pollution appear to be species‐specific. No differences in microsatellite heterozygosity or allele number were found between a control site and two sites suffering from cadmium/zinc pollution in bullhead (*Cottus gobio*; Knapen et al., 2009). Similarly, McMillan, Bagley, Jackson, and Nacci (2006) found no differences in diversity, as measured by AFLP markers, in populations of Atlantic killifish (*Fundulus heteroclitus*) that differed in their exposure to polychlorinated biphenyls (PCBs).

The evidence of genetic erosion, either through the action of mining practices, barriers or a combination of both, in several of the rivers and streams studied here raises concerns as to the long‐term genetic viability of these populations. The eroded populations are all within small catchments that display high levels of relatedness and homozygosity (at least at the loci studied here). Genetic erosion has serious negative effects at the population level, over both short and long timescales and could potentially impede adaptive responses of small populations to future stressors (Bijlsma & Loeschcke, 2012; Willi, Buskirk, & Hoffmann, 2006). For instance, it is apparent that the rivers of southern Britain are becoming warmer (Jonkers & Sharkey, 2016) and that increasing summer temperatures in particular have been implicated in declines in trout populations (Clews, Durance, Vaughan, & Ormerod, 2010); thus, it is likely that the upper thermal tolerance limits for trout (19.5°C) will be locally exceeded within the next few decades (Jonkers & Sharkey, 2016).

Where small streams have been relatively unaffected by mining activities and where migration barriers are absent, trout populations exhibit levels of genetic diversity and divergence that are comparable to the larger rivers in the region. The Caerhays is found to the east of the Carrick Roads and the trout within it appear genetically very similar to those in the Fal, Allen, and Tresillian. Similarly, the Lerryn is a small stream that enters the lower reaches of the Fowey estuary and the fish within it do not appear distinct from those of the Fowey and both the West and East Looe rivers. For both streams, there are no obvious barriers to fish movement in their lower reaches. This suggests that in the absence of barriers to movement, trout in these rivers and streams may constitute metapopulations, with the small stream populations being able to maintain levels of diversity via gene flow with trout in the larger catchments.

The significant predicted loses of heterozygosity and the high levels of relatedness, along with fact that genetic drift is a dominant evolutionary force, show that trout in small, isolated rivers are in danger of further severe reductions in diversity and heterozygosity. Such decreases have serious consequences for the future survival of these populations. For endangered species, reductions in heterozygosity can lower their evolutionary potential (e.g., their ability to cope with future climate change), compromise their reproductive fitness and elevate the risk of extinctions (Spielman et al., 2004). These generalizations apply equally to small, isolated populations of nonendangered species, such as those highlighted here. Five fitness‐related traits were significantly correlated to levels of heterozygosity in the three‐spined stickleback (*Gasterosteus aculeatus*), while there was an increased risk of extinction in populations of the Glanville fritillary (*Melitaea cinxia*) with reduced heterozygosity (Lieutenant‐Gosselin & Bernatchez, 2006; Saccheri et al., 1998). Additionally, large outbreeding populations of lake trout (*Salvelinus namaycush*) have lower numbers of deleterious mutations than smaller, more inbred populations, highlighting that purifying selection may be less effective in such populations (Perrier et al., 2017). Together, all these factors suggest that reductions in levels of diversity and heterozygosity may put small, isolated trout populations at risk of extinction before demographic changes become apparent (Spielman et al., 2004).

We have highlighted that human activities over long timescales have affected the structuring of, and the levels of genetic diversity within, trout populations inhabiting streams and rivers of varying sizes. While all populations analyzed appear to have gone through severe genetic bottlenecks, the small stream populations have been disproportionately affected. Despite this, the small, isolated populations still possess unique, private markers. The neutral microsatellite markers utilized here, however, do not give us any indication of whether diversity has similarly been reduced in loci of adaptive significance, for example, major histocompatibility (MHC) gene loci. It is also clear that some of the populations from the smallest streams are isolated from the main trout metapopulation along this stretch of coast. Future conservation efforts should investigate ways of increasing genetic diversity within the outlier populations, preferably by enabling natural reconnection with fish inhabiting proximal larger catchments.

## CONFLICT OF INTEREST

None declared.

## AUTHOR CONTRIBUTION

**R. Andrew King:** Data curation (lead); Formal analysis (lead); Visualization (lead); Writing‐original draft (equal); Writing‐review & editing (equal). **Jamie R. Stevens:** Conceptualization (equal); Funding acquisition (equal); Supervision (lead); Writing‐original draft (equal); Writing‐review & editing (equal). **Bruce Stockley:** Conceptualization (equal); Funding acquisition (equal); Writing‐original draft (supporting); Writing‐review & editing (supporting). 

## Data Availability

Microsatellite genotypes are available through the Dryad repository at https://doi.org/10.5061/dryad.4b8gtht8w.

## APPENDIX 1

**Table A1:** Details of catchment area, stream length and Strahler Stream Order for the 16 south Cornish rivers studied; rivers with genetic outlier trout populations are marked *

River	Area (km^2^)	Stream length (km)	Strahler stream order
Helford*	14.604	8.496	2
Kennall*	32.317	18.057^a^	4
Allen	29.197	15.331	4
Fal	108.243	53.043	4
Tresillian	58.152	29.138	4
Percuil*	6	5.945	1
Caerhays	28.767	13.677	3
Portmellon*	7.081	2.83	3
Par*	57.808	28.993	4
Fowey	174.614	83.9	4
Lerryn	24.3	11.665	3
Polperro*	13.136	6.945	3
West Looe	48.709	17.46	4
East Looe	45.22	23.605	4
Seaton	56.224	27.363	4
Lynher	151.498	57.548	4

Accessible stream length is <5 km due to the presence of an impassable weir system situated within an historic gunpowder works at Kennall Vale dating from the 19th century.

**Table A2:** Details of number of full sib families and number of individuals per family, as determined using COLONY, found in trout sampled from 26 sites in Cornwall

Site	Code	Full sib families	Family size	Site	Code	Full sib families	Family size
1	HEL.MER	3	2,2,2	14	FOW.CAR	1	4
2	KEN.TRE	4	2,2,2,4	15	FOW.LES	2	2,3
3	KEN.PON	3	2,3,3	16	FOW.TRE	1	2
4	ALL.DAU	2	2,3	17	FOW.WAR	4	2,2,2,3
5	FAL.TGY	3	2,2,3	18	LER.COL	1	2
6	FAL.TRE	2	2,2	19	POL.LON	3	2,2,5
7	TRE.GEE	3	2,2,2	20	WLO.GIL	1	2
8	PER.TRE	2	2,2	21	ELO.HIG	2	2,2
9	CAE.KIL	2	2,2	22	SEA.COU	1	2
10	POR.GAL	5	2,3,4,4,4	23	SEA.HES	1	2
11	PAR.BRI	2	2,2	24	LYN.BAT	4	2,2,2,3
12	FOW.BUL	6	2,2,2,2,2,3	25	LYN.KER	4	2,2,2,3
13	FOW.CAB	3	2,3,4	26	LYN.KNI	2	2,2

**Table A3:** Results from Bottleneck (Piry et al., 1999) and M‐ratio (Garza & Williamson, 2001) for the 16 south Cornish rivers studied

River/stream	pTPM	Mode shift	M	Critical M (Mc)				
				Θ = 0.1	Θ = 0.2	Θ = 0.5	Θ = 1	Θ = 2
Helford	0.2706	Normal L‐shaped	0.5195	0.8036	0.7932	0.7694	0.7399	0.7168
Kennall	0.7910	Normal L‐shaped	0.6090	0.8011	0.7909	0.7663	0.7455	0.7274
Allen	0.6746	Normal L‐shaped	0.6213	0.7994	0.7902	0.7662	0.7440	0.7193
Tresillian	0.6603	Normal L‐shaped	0.6546	0.8015	0.7926	0.7692	0.7438	0.7231
Fal	0.3690	Normal L‐shaped	0.7114	0.8036	0.7921	0.7695	0.7463	0.7323
Percuil	0.5391	Normal L‐shaped	0.5114	0.8031	0.7949	0.7685	0.7444	0.7206
Caerhays	0.6746	Normal L‐shaped	0.6307	0.8029	0.7937	0.7687	0.7443	0.7225
Portmellon	0.2576	Normal L‐shaped	0.4967	0.8000	0.7913	0.7652	0.7391	0.7151
Par	0.9479	Normal L‐shaped	0.5209	0.8028	0.7924	0.7690	0.7417	0.7180
Fowey	0.6160	Normal L‐shaped	0.7589	0.8038	0.7931	0.7722	0.7545	0.7460
Lerryn	0.2706	Normal L‐shaped	0.6788	0.8038	0.7926	0.7708	0.7438	0.7215
Polperro	0.0001	Normal L‐shaped	0.5437	0.8012	0.7933	0.7667	0.7434	0.7203
West Looe	0.4144	Normal L‐shaped	0.6774	0.8009	0.7926	0.7688	0.7441	0.7223
East Looe	0.1377	Normal L‐shaped	0.6705	0.8020	0.7931	0.7692	0.7441	0.7218
Seaton	0.4765	Normal L‐shaped	0.6089	0.8029	0.7926	0.7676	0.7473	0.7295
Lynher	0.3840	Normal L‐shaped	0.7105	0.8029	0.7926	0.7703	0.7498	0.7352

pTPM—the probability of a bottleneck under the two‐phase model from Bottleneck. M—ratio of the number of alleles at a given microsatellite locus to the allelic size range. Critical M was calculated based on pre‐bottleneck effective population sizes of 50, 100, 250, 500 and 1,000.

**Table A4:** Predicted future reductions in observed heterozygosity (H_O_), based on Crow & Kimura (1970)

River	N_E _(LD)	Ne (sibship)	Current H_O_	Predicted H_O_ *n* generation in future			Percentage loss in H_O_ *n* generation in future		
				10	50	100	10	50	100
Helford	223.3	54	0.654	0.640/0.596	0.585/0.411	0.523/0.258	2.22/8.83	10.60/37.19	20.08/60.55
Kennall	162.6	114	0.712	0.690/0.681	0.610/0.571	0.523/0.459	3.03/4.30	14.27/19.73	26.51/35.57
Allen	1748.1	98	0.763	0.760/0.725	0.752/0.591	0.741/0.457	0.29/4.97	1.42/22.57	2.82/40.04
Tresillian	524.1	98	0.756	0.748/0.718	0.720/0.585	0.687/0.453	0.95/4.97	4.66/22.57	9.10/40.04
Fal	1643.6	141	0.781	0.779/0/754	0.769/0.654	0.758/0.547	0.30/3.49	1.51/16.27	3.00/29.90
Percuil	90.3	44	0.620	0.586/0.553	0.469/0.350	0.356/0.198	5.4010.80	24.24/43.53	42.61/68.11
Caerhays	349.3	107	0.746	0.735/0.711	0.694/0.590	0.646/0.467	1.42/4.58	6.91/20.88	13.35/37.40
Portmellon	59.7	29	0.679	0.624/0.570	0.446/0.284	0.293/0.119	8.07/15.96	34.33/58.09	56.87/82.43
Par	159.2	59	0.693	0.672/0.636	0.592/0.453	0.506/0.296	3.10/8.16	14.55/34.66	26.99/57.30
Fowey	567	293	0.779	0.7720.765	0.745/0.715	0.713/0.656	0.88/1.69	4.32/8.19	8.44/15.70
Lerryn	419.7	92	0.782	0.773/0.740	0.737/0.595	0.694/0.453	1.18/5.30	5.79/23.85	11.24/42.01
Polperro	121.5	57	0.761	0.730/0.697	0.619/0.490	0.504/0.315	4.04/8.43	18.63/35.63	33.79/58.57
West Looe	311.1	109	0.790	0.777/0.754	0.729/0.625	0.673/0.495	1.60/4.58	7.73/20.88	14.86/37.40
East Looe	751.2	96	0.809	0.804/0.768	0.783/0.623	0.757/0.480	0.66/5.09	3.27/22.98	6.44/40.68
Seaton	396.2	109	0.779	0.769/0.744	0.731/0.619	0.687/0.492	1.25/4.94	6.12/20.54	11.86/36.86
Lynher	935.9	230	0.781	0.7770.764	0.760/0.700	0.740/0.628	0.53/2.15	2.64/10.31	5.20/19.56

Calculations are based on current H_O_ and effective population size (N_E_) estimated using the linkage disequilibrium (LD) method as implemented in NEESTIMATOR v.2 (Do et al., 2014), and the sibship method as implemented in COLONY v 2.0.5.9 (Jones & Wang, 2010). Predicted future H_O_ and predicted percentage loss in H_O_ are given as LD method/sibship method.
